# Survival of Children With Pulmonary Embolism Supported by Extracorporeal Membrane Oxygenation

**DOI:** 10.3389/fped.2022.877637

**Published:** 2022-05-03

**Authors:** John S. Kim, Cindy S. Barrett, Robert W. Hyslop, Shannon M. Buckvold, Katja M. Gist

**Affiliations:** ^1^Department of Pediatrics, Heart Institute, Children’s Hospital Colorado, University of Colorado School of Medicine, Aurora, CO, United States; ^2^ECMO Department, Heart Institute, Children’s Hospital Colorado, Aurora, CO, United States; ^3^Division of Cardiology, Department of Pediatrics, Cincinnati Children’s Hospital Medical Center, University of Cincinnati, Cincinnati, OH, United States

**Keywords:** pulmonary embloism, ECMO - extracorporeal membrane oxygenation, database, survival, children

## Abstract

The purpose of this study was to describe the demographics and in-hospital mortality of children (<18 years) from 2007 to 2018 supported by Extracorporeal Membrane Oxygenation (ECMO) for a primary diagnosis of pulmonary embolism and reported to the Extracorporeal Life Support Organization database. Fifty-six patients were identified and 54 were included in this analysis. A total of 33 patients (61%) survived. No differences in demographics or ECMO details (duration, mode, and support type) were found between survivors and non-survivors. When ECMO complications were compared, pulmonary bleeding occurred more frequently in non-survivors (23.8%, *n* = 5) compared to survivors (*n* = 0) (*p* = 0.006).

## Introduction

Pulmonary embolism (PE) and venous thromboembolism (VTE) are major causes of mortality, morbidity, and hospitalization worldwide. The estimated incidence of PE and VTE in ranges from 100–200 per 100,000 adults and 1.4–1.9 per 100,000 children (1, 2). Reviews of the Pediatric Health Information System (PHIS) data report an incidence of 34–58 cases of VTE per 10,000 pediatric hospital admissions (2, 3). Mortality associated with PE in children is estimated at 8.3% (4). Extracorporeal membrane oxygenation (ECMO) is described as a useful means of supporting adults with massive PE with survival reported between 40 and 60% (5–9). Recent reports have demonstrated the successful rescue of children with massive PE using ECMO (6).

In this study, we aim to describe the demographics and in-hospital mortality for pediatric patients (age < 18 years) supported on ECMO for PE. We hypothesized that ECMO support for PE would be associated with greater than 50% survival.

## Materials and Methods

We queried the Extracorporeal Life Support Organization (ELSO) database for children <18 years of age who received ECMO support between 2007 and 2018 with a primary ICD-9 (415.x) or ICD-10 (I26.x) diagnostic code of PE (10). This study was approved by the ELSO Registry Scientific Oversight Committee. Demographics, ECMO support details, and ECMO complications were assessed. Patients with missing survival data or >1 ECMO run were excluded. Our primary study outcome was survival to hospital discharge. Descriptive statistics were performed and reported using median with interquartile range and proportions for continuous and categorical variables as appropriate. Variables were compared using Mann–Whitney U test or Chi Square/Fisher exact tests as appropriate.

## Results

**FIGURE 1:**
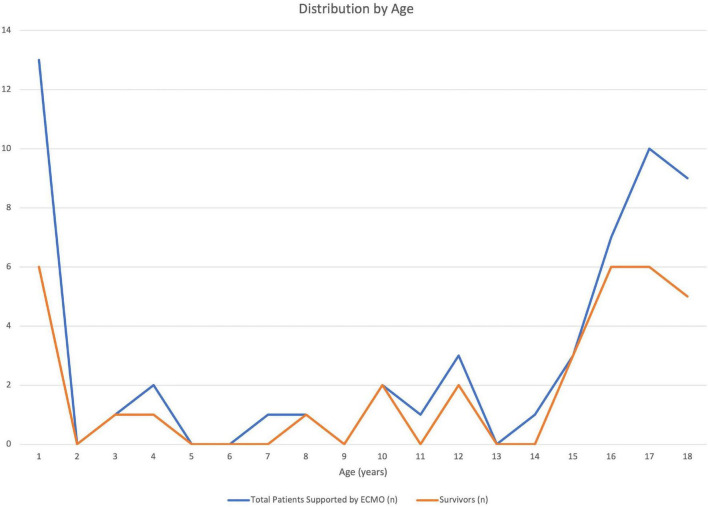
Distribution of patients in the Extracorporeal Life Support Organization (ELSO) database with a primary diagnosis of pulmonary embolism by age during the study period (2007–2018). Total patients and survivors (n) displayed by year of life.

A total of 56 patients met inclusion criteria, 2 patients were excluded due to missing survival data and second run. Thirty-three patients (61%) survived to hospital discharge. There was no difference in demographics (age, sex, weight, and race) between survivors and non-survivors. Thirteen of the 54 patients were infants less than 1 year and 30 patients were adolescents over 12 years of age. Figure 1 displays the distribution of number of patients by age (total and survivors). ECMO complications were compared between the two groups and pulmonary bleeding was found to be more common in non-survivors (23.8%, *n* = 5) compared to survivors (*n* = 0) (*p* = 0.006) (Table 1). In addition, though significance was not reached, there was a trend toward more CNS and ECMO site bleeding in non-survivors (Table 1). A multivariable analysis was not able to be performed due to model instability.

**TABLE 1:** Demographics and complications.

Variable	Overall (*n* = 54)	Survivors (*n* = 33)	Non-Survivors (*n* = 21)	*P*-value
Age (years)	14.9 (2.3, 16.6)	15.1 (5.5, 16.6)	11.9 (0.2, 17.8)	0.50
Age 0 to 30 d (n) Age 31 d to 1 yr (n) Age 1 to 12 yr (n) Age 12 to 18 yr (n)	7 6 11 30	3 3 7 20	4 3 4 10	N/A
Sex, male (missing *n* = 1)	20 (37.8)	14 (42.4)	6 (28.6)	0.39
Weight (kg)	58.2 (19.4, 85.5)	62.9 (24.9, 89.2)	51.5 (7.35, 84.0)	0.39
Race White Black Other	30 (55.6) 11 (20.4) 13 (24.1)	18 (54.5) 7 (21.2) 8 (24.2)	12 (57.1) 4 (19.0) 5 (23.8)	0.71
ECMO duration (hrs) (missing *n* = 1)	91 (26, 183)	120 (47, 184)	48 (4, 183)	0.09
Mode (VA)	51 (94.4)	30 (90.9)	21 (100)	0.27
Support type Cardiac eCPR Pulmonary	20 (37.0) 13 (24.1) 21 (38.9)	11 (33.3) 7 (21.2) 15 (45.5)	9 (42.9) 6 (28.6) 6 (28.6)	0.52
Year ECLS 2007–2012 2013–2018	21 (38.9) 33 (61.1)	21 (63.6) 12 (36.4)	9 (42.9) 12 (57.1)	0.78
ECMO Complications AKI with RRT Infection (culture+) Bleeding CNS Bleeding site Bleeding GI Bleeding pulmonary Bleeding Tamponade Mechanical	14 (25.9) 6 (11.1) 3 (5.6) 17 (31.5) 4 (7.4) 5 (9.3) 2 (3.7) 17 (31.5)	9 (27.3) 3 (9.1) 0 (0) 7 (21.2) 1 (3.0) 0 (0) 2 (6.1) 9 (27.3)	5 (23.8) 3 (14.3) 3 (14.3) 10 (47.6) 3 (14.3) 5 (23.8) 0 (0) 8 (38.1)	1.00 0.67 0.05 0.07 0.29 0.006 0.52 0.55

*Continuous variables presented as median with IQR and compared using non-parametric Mann–Whitney U tests.*

*Categorical variables presented as n (%) and compared using chi squared or fisher exact tests as appropriate.*

## Discussion

Pulmonary embolism is a rare disease that contributes to significant morbidity in hospitalized children. The requirement of ECMO support associated with a diagnosis of PE is further associated with significant mortality. Interestingly, it appears that only a small number of children are supported by ECMO for PE but the bimodal distribution of PE is maintained with a propensity to infants and adolescents. Given the small sample size, we were not able to study institutional practices (e.g., some programs in ELSO do not support children with VV ECMO) or evaluate illness severity prior to and associated with mode selection. However, this study suggests that bleeding complications may be associated with non-survival (pulmonary bleeding was more common in the non-survivor group with similar trends in other bleeding complications). This is of particular importance in this context of necessary anticoagulation (and possible fibrinolytic therapy) for treatment of PE. It is also possible, that emerging anticoagulation strategies may demonstrate improved bleeding risk profiles, thus reducing the possible morbidity and mortality associated with ECMO support for ECMO. ECMO support in the setting of PE in children is promising (6), however, limited data in children make it difficult to recommend its widespread use. Nevertheless, our evaluation of the ELSO database demonstrated survival of 61% in children, similar to other published studies reporting an approximate adult survival of 40–60% (7–9). Thus, ECMO may be a reasonable means for supporting children with massive PE. Further research to develop standardization of treatment and to understand the use of ECMO for PE is needed.

The study has several limitations. As only 54 patients were identified from the ELSO database in a 12-year period, our study cohort was small and limited our statistical evaluation. PE in children is a rare problem with very few children requiring ECMO. This study utilized data from a database of children supported by ECMO from centers that participate in ELSO and there are limitations in the data available, including data concerning medical decision-making. In addition, analysis of the ELSO database will not include children supported by ECMO at non-participating centers. Data describing center characteristics and practices were not captured in our data, therefore, the influence of these factors cannot be evaluated. In particular, we could not evaluate the anticoagulation practices or fibrinolytic therapies utilized in these patients with PE. It is also possible that the primary diagnosis of PE was underestimated in the ELSO database. Although our query identified patients with a primary ICD code for PE associated with ECMO support, we could not definitively identify whether the primary indication for ECMO support was PE, nor did we evaluate PE as a complication of ECMO. Patients included in the study were screened by identification with ICD-9 and ICD-10 codes, the coding of which is dependent on the accuracy of the participant centers. Finally, we could not evaluate the impact of different anticoagulant and thrombolytic therapies.

## Conclusion

In conclusion, our study was able to demonstrate a favorable survival of 61% for children with PE who are supported by ECMO. Non-survivors were more likely to have a pulmonary hemorrhage (when compared to no reported pulmonary hemorrhage in the survivors). Further investigations concerning management of patients with PE who require ECMO support are needed.

## Data Availability Statement

The original contributions presented in the study are included in the article/supplementary material, further inquiries can be directed to the corresponding author.

## Author Contributions

JK is primarily responsible for the database request, data analysis, data interpretation, and manuscript preparation. CB, RH, and SB contributed to the study design, data interpretation, and manuscript revision. KG contributed to the study design, data analysis, data interpretation, and manuscript preparation. All authors contributed to the article and approved the submitted version.

## Conflict of Interest

The authors declare that the research was conducted in the absence of any commercial or financial relationships that could be construed as a potential conflict of interest.

## Publisher’s Note

All claims expressed in this article are solely those of the authors and do not necessarily represent those of their affiliated organizations, or those of the publisher, the editors and the reviewers. Any product that may be evaluated in this article, or claim that may be made by its manufacturer, is not guaranteed or endorsed by the publisher.
